# Leak Event Diagnosis for Power Plants: Generative Anomaly Detection Using Prototypical Networks

**DOI:** 10.3390/s24154991

**Published:** 2024-08-01

**Authors:** Jaehyeok Jeong, Doyeob Yeo, Seungseo Roh, Yujin Jo, Minsuk Kim

**Affiliations:** 1 Department of Electronic Information System Engineering, Sangmyung University, Cheonan 31066, Republic of Korea; 2 Nuclear System Integrity Sensing and Diagnosis Division, Korea Atomic Energy Research Institute, Daejeon 34057, Republic of Korea; 3 Department of Human Intelligence and Robot Engineering, Sangmyung University, Cheonan 31066, Republic of Korea

**Keywords:** GAD-PN, anomaly detection, CycleGAN, prototypical networks

## Abstract

Anomaly detection systems based on artificial intelligence (AI) have demonstrated high performance and efficiency in a wide range of applications such as power plants and smart factories. However, due to the inherent reliance of AI systems on the quality of training data, they still demonstrate poor performance in certain environments. Especially in hazardous facilities with constrained data collection, deploying these systems remains a challenge. In this paper, we propose Generative Anomaly Detection using Prototypical Networks (GAD-PN) designed to detect anomalies using only a limited number of normal samples. GAD-PN is a structure that integrates CycleGAN with Prototypical Networks (PNs), learning from metadata similar to the target environment. This approach enables the collection of data that are difficult to gather in real-world environments by using simulation or demonstration models, thus providing opportunities to learn a variety of environmental parameters under ideal and normal conditions. During the inference phase, PNs can classify normal and leak samples using only a small number of normal data from the target environment by prototypes that represent normal and abnormal features. We also complement the challenge of collecting anomaly data by generating anomaly data from normal data using CycleGAN trained on anomaly features. It can also be adapted to various environments that have similar anomalous scenarios, regardless of differences in environmental parameters. To validate the proposed structure, data were collected specifically targeting pipe leakage scenarios, which are significant problems in environments such as power plants. In addition, acoustic ultrasound signals were collected from the pipe nozzles in three different environments. As a result, the proposed model achieved a leak detection accuracy of over 90% in all environments, even with only a small number of normal data. This performance shows an average improvement of approximately 30% compared with traditional unsupervised learning models trained with a limited dataset.

## 1. Introduction

Automated anomaly detection systems for preemptively monitoring safety incidents are considered essential technologies, as their effectiveness has been demonstrated in diverse fields. In these systems, methods for automatically distinguishing anomalies typically include a variety of approaches, including statistical techniques and rule-based methods. Recently, artificial intelligence (AI) systems, which are training neural networks, have gained considerable attention for achieving high detection performance [1,2,3].

Traditional AI-based machine learning approaches for anomaly detection systems can be divided into supervised and unsupervised learning. In supervised learning-based systems, labeled normal and abnormal data are used to train models that classify between abnormal and normal states. It is typical to demonstrate high accuracy and benefits in differentiating between normal and abnormal states. However, in practice, obtaining abnormal data can be challenging, which has led to the adoption of unsupervised learning-based anomaly detection systems.

The unsupervised learning method trains the model using only normal data. Then, when new data are provided, the system evaluates the degree to which the data deviate from the learned normal patterns to detect anomalies. Due to these advantages, this method is currently applied in most anomaly detection systems. For instance, sparse Bayesian learning (SBL) models use a probabilistic data-driven method for structural damage detection in structural health monitoring (SHM) [4]. This approach effectively detects structural damage and allows for quick analysis due to its simple structure. In addition, the enhanced hierarchical sparse Bayesian learning (eHSBL) [5] model uses Gaussian kernels and hierarchical Bayesian models to accurately model heteroscedastic data in high-dimensional spaces, quantify uncertainties, and analyze correlations with high accuracy, even in extreme situations such as typhoons. This makes it advantageous for high-accuracy data modeling, uncertainty quantification, and correlation analysis in SHM measurements. However, there are still problems in certain environments. The performance of unsupervised learning-based anomaly detection systems is significantly influenced by large-scale, high-quality data. Consequently, it is challenging to apply these systems in hazardous facilities such as power plants and research laboratories, where data collection is difficult. To address these data constraints, some methods utilize simulation data [6,7,8]. However, the data collection in the simulation environment can cause differences between real domains, and it is very difficult to synchronize all environmental information and parameters for the sensors with real environments. Furthermore, anomaly detection models trained on comparable environments or simulated data may experience reduced accuracy when deployed in real-world settings.

Recently, research in meta-learning-based anomaly detection has also aimed to bridge the gap between simulation data and real-world environmental domains [9,10]. Meta-learning is a learning method that enables AI-based anomaly detection models to rapidly adapt to new environments, using an approach called “learn-to-learn” [11]. In other words, the model learns how to learn in different environments, allowing it to learn effectively with only a small number of data. MAML [12] and Prototypical Networks (PNs) [13] are two representative methods of meta-learning. The MAML-based approach involves training the initial parameters of a model’s neural network to rapidly adapt to new domains. It provides the advantage of seamless integration with existing supervised and unsupervised AI methods. However, unsupervised learning-based anomaly detection, which requires extended training periods and utilizes only normal data, may struggle to accurately capture the distribution of normal data. PNs represent a supervised learning approach to categorize anomalies by measuring distances and similarities between class prototypes and new data in the learned embedding space. Specifically, PNs generate a prototype for each class using *n* support data points. Once query data are entered, the model can be updated to compare similarities with these prototypes and to maximize similarities with the same class. By using metadata from various environments, it becomes possible to rapidly adapt to a new environment with limited data. Moreover, it achieves high anomaly classification performance with only a few abnormal data. However, it still requires some abnormal data, making it difficult to apply in environments where such data are difficult to collect.

**Figure 1:**
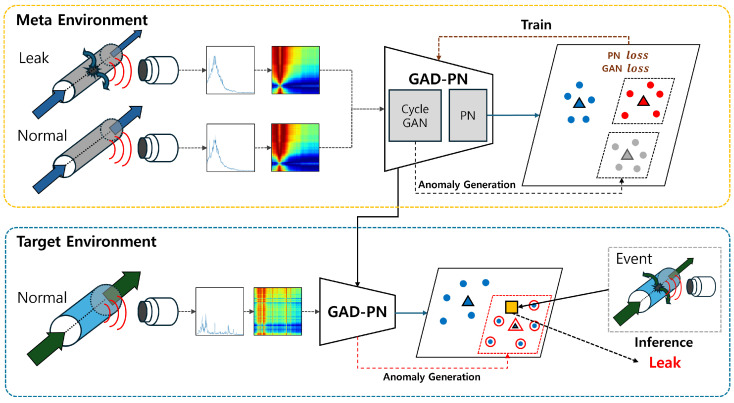
Overview of the GAD-PN for pipe leak detection.

In this paper, we proposed a Generative Anomaly Detection using Prototypical Networks (GAD-PN) framework designed to detect anomalies using a small number of normal data. The method adopts CycleGAN [14] to generate abnormal data from normal data, which are subsequently used by PNs for anomaly detection. Based on the proposed generative model, we make the following assumption: if *A* and *B* are normal data, and $A_{anomaly}$ and $B_{anomaly}$ are abnormal data, then $C_{anomaly}$ can be generated from *C* by leveraging the characteristic ‘anomaly’. Based on this assumption, we train the CycleGAN model using data that closely reflect the characteristics of the target deployment environment. This allows PNs to generate normal and abnormal prototypes to perform inference using only normal data. In addition, in this paper, numerical sensor time series (TS) data are converted into images via a Gramian angular field (GAF) transformation for pre-processing. By leveraging the robust feature extraction capabilities of Convolutional Neural Networks (CNNs), we can achieve higher accuracy with image data compared with TS data. The overview of our proposed structure is shown in Figure 1. This approach effectively resolves the challenges associated with collecting normal and abnormal data in real-world environments, as well as mitigating the complexities arising from environmental differences.

The main contributions of this paper can be summarized as follows:

- GAD-PN is designed to enable anomaly detection in environments such as hazardous facilities by using just a small number of normal data from the target domain, where data collection is limited.
- Our method leverages metadata and CycleGAN to learn the features necessary for transforming normal data into abnormal data within the target domain, thereby enhancing adaptability to different environments.
- By leveraging the meta-learning model PN to acquire the capability to distinguish between normal and abnormal data (learn-to-learn), we realize high accuracy in anomaly detection with only a limited dataset from the target domain.

## 2. Related Works

### 2.1. Traditional Anomaly Detection

In general, traditional image-based anomaly detection models rely primarily on unsupervised learning using only normal data, considering the difficulty of obtaining abnormal data. PatchCore [15] is a model inspired by SPADE [16] and PaDIM [17], performed by training exclusively on normal images for anomaly detection. Normal images are segmented into patches, and features are extracted using a pre-trained encoder, which is subsequently stored in a memory bank. Anomaly detection is performed by using the distance between these stored features and the features extracted from the input image, achieving more efficient training and higher performance compared with previous works. As a result, models such as OpenPatch [18] and DDAD [19], which adopt this approach, have emerged and achieved high performance, surpassing 99% area under receiver operator characteristic curve (AUROC) on the MVTec [20] anomaly detection dataset. However, traditional anomaly detection methods require a large number of data for initial training, which makes it difficult to achieve good performance with only a small number of samples available. In real-world environments it is often difficult to collect data, and meta-learning-based anomaly detection methods that perform anomaly detection with a small number of samples have been proposed to overcome these problems.

### 2.2. Meta-Learning-Based Anomaly Detection

In meta-learning-based unsupervised learning approaches, research has explored adopting the training method known as MAML. This has the advantage of being easy to apply to models based on gradient descent by learning initial parameters that adapt quickly. It is easily combinable with existing traditional unsupervised anomaly detection models, and recently MAVAE [21] has been proposed. MAVAE uses variational autoencoders (VAEs) [22] to train the initial parameters of the VAE using the MAML method. Subsequently, during the inference process, the VAE is fine-tuned with a small number of normal data, enabling effective anomaly detection, even with limited data. Most research on meta-learning-based supervised learning approaches has adopted the PN method. FSL-PN [23], which applies contrastive loss, or PRN [24], which introduces a method for reconstructing anomaly segmentation maps using a multi-scale self-attention module, have been proposed. Each model demonstrated stability and high detection performance during training, but they require labeled anomaly data. In this paper, we assume that leakage characteristics in different environments are similar in the context of plant pipeline leakage scenarios. Therefore, by integrating a generative model capable of domain transformation with PNs in the GAD-PN structure, high detection performance can be achieved with the input of only a small number of normal data input.

### 2.3. Time Series-to-Image Translation

Traditionally, anomaly detection in time series data has been approached using models based on recurrent neural networks (RNNs) and long short-term memory (LSTM). Several models have combined RNNs and autoencoders to detect anomalies based on reconstruction errors [25]. However, these methods, while effective for time series analysis, do not support parallel processing of input data and incur high computational costs. There is also the problem that long-term dependencies are not reflected or anomalous patterns are not properly recognized. To resolve these problems, specific labeling of anomalous parts of the time series data was performed to improve anomaly pattern recognition performance [26,27]. However, labeling each time series data point individually requires significant resources. To address these challenges, recent studies have focused on converting time series data into images for anomaly detection.

An anomaly detection algorithm using images simplifies the process by requiring labeling only for images converted from a single sequence of time series data. Additionally, it is able to detect advanced anomaly patterns using CNNs. In [28], to detect myocardial infarction (MI), electrocardiogram (ECG) signals were converted into a GAF for anomaly detection. The results demonstrate that converting ECG signals into GAF images for MI detection is effective. In [29], experiments for fluid prediction transformed one-dimensional time series data into two-dimensional matrix representations using GAF transformations. Based on this approach, the CNN model effectively captured the nonlinear structures and patterns present in the time series data, resulting in enhanced accuracy and stability for predicting fluid behavior. In [30], anomaly detection in the manufacturing field was difficult due to the lack of labels and the imbalance of the time series data obtained from the manufacturing process. To overcome this, the GAF encoding method was used to improve the performance of anomaly detection. Therefore, in this paper, we adopt GAF transformations on pipe leakage datasets collected in TS format and adopt CNN-based models. Consequently, this enhances anomaly detection and anomaly data generation models by improving anomaly pattern recognition and enhancing the model’s generalization performance.

## 3. Analysis of Piping Leak Dataset in Operational Power Plants

### 3.1. Data Collection and Analysis of a Piping Leakage

This paper reproduced and collected leakage scenario data for plant piping. In the leak situation, ultrasonic signals in the range of 20 kHz to 100 kHz were collected following the ASTM E 1002-11 standard [31] of Table 1 to detect leaks. The low-power wireless ultrasound sensor module of [32] was used to collect signals for leakage detection. In general, ultrasound signals attenuate as distance increases, thus requiring amplification. Therefore, the analog sound signal collected through the microphone was amplified using an amplifier and converted into a digital signal via an analog-to-digital converter (A/D converter) at a sampling frequency of 256 kHz. In this case, each average spectrum is represented as a 320-dimensional vector.

### 3.2. Dataset Based on Pipe Leakage Scenario

**Figure 2:**
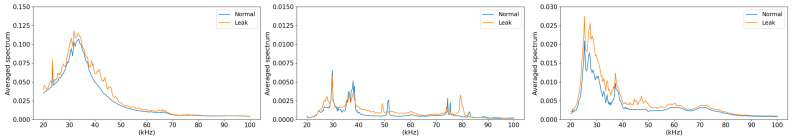
Plot results of pipe leak scenario: (**Left**) A, (**Middle**) B, (**Right**) C.

Based on the data collection environment described in Section 3.1, we collected the experimental dataset in this paper. To collect diverse datasets based on different experimental environments as described in Section 3.1, data were collected from three experimental environments, labeled A, B, and C, in which parameters such as the fluid pressure and hole size of the pipe were set differently. Depending on the features of the data, they can be analyzed and collected using methods such as autocorrelation functions, correlation functions, time-frequency patterns size analysis of measurement data, etc. The dataset includes normal and leakage data collected for learning and evaluation according to the time/frequency domain and the features of the ultrasonic signal. The average representations of normal and leakage data in the scenario are shown in Figure 2.

From the left to right, the figure describes A, B, and C, with normal data represented by blue lines and leakage data by orange lines. Through the results, it can be confirmed that there is a clear distinction between normal and leakage conditions, with each environment exhibiting distinct patterns. The number of data points for each scenario is as shown in Table 2.

## 4. Materials and Methods

**Figure 3:**
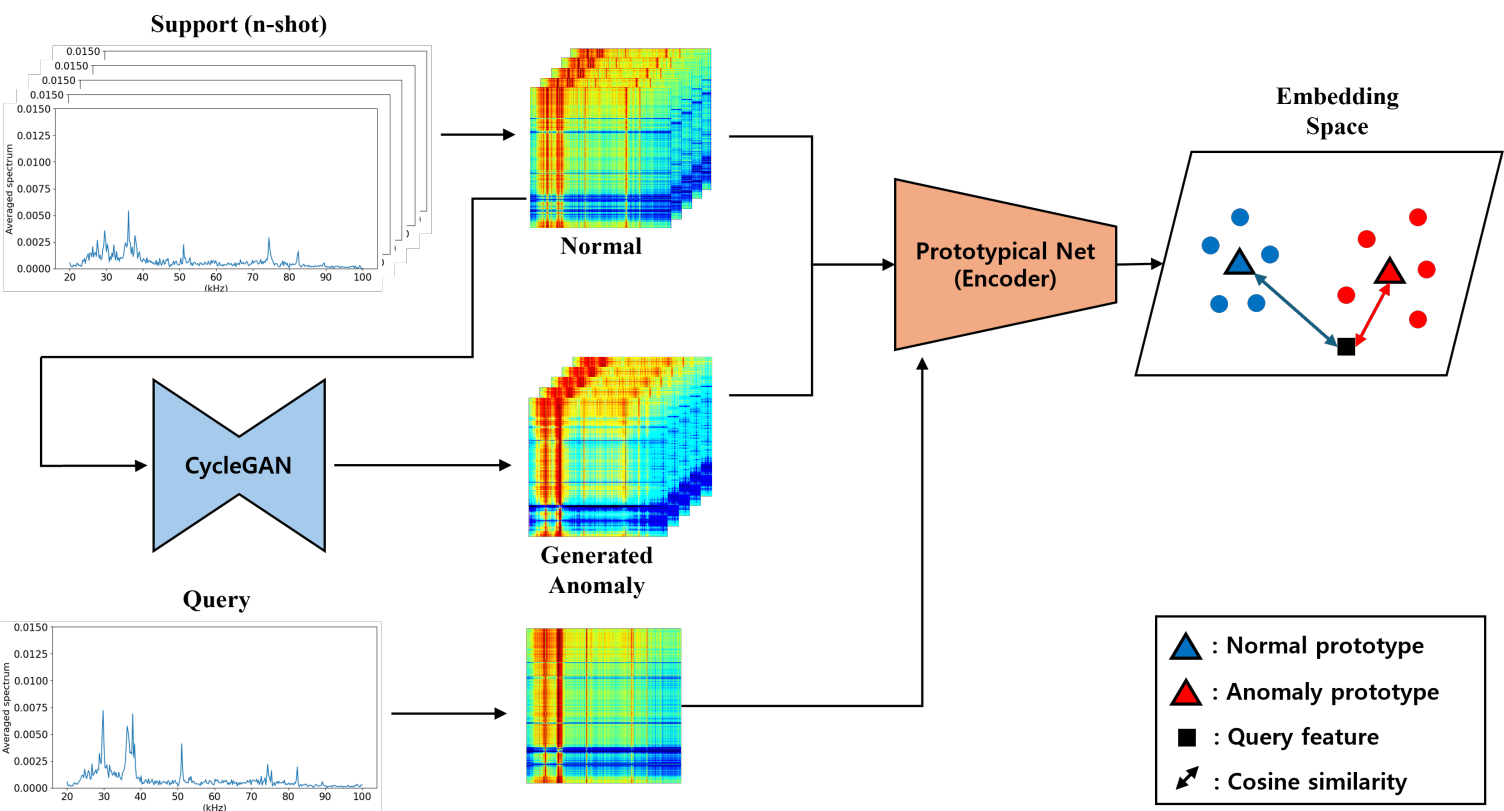
Inference process of anomaly generation over the prototypical network.

In this section, we propose a method using GAD-PN for pipe leak monitoring. GAD-PN receives a small number of normal data, converts them into an image, and then generates leakage data based on CycleGAN. With the generated data, the PN creates prototypes of normal and leak states in the embedding space. Then, using the query data for inference, the cosine similarity between each prototype is calculated, and the data are classified as either in a state of normal or leak. The inference procedure of GAD-PN is shown in Figure 3. The subsections below detail the components of GAD-PN. Section 4.1 presents the process of transforming TS signal data into 2D images using the GAF transformation. Section 4.2 describes the method of generating leak data from normal data using CycleGAN. Lastly, Section 4.3 discusses the method of training the PN using metadata to apply its adaptation to new domains.

### 4.1. Time Series Data to 2D Image Using GAF Transformation

The data converted to images can be used to recognize anomalies in time patterns as spatial patterns using CNNs. In general, CNNs can extract local features of the input data via convolution operations and learn them in complex patterns due to their hierarchical structure. For this reason, CNNs have an advantage in anomaly detection due to their high pattern recognition capability. By employing CNNs that can have the characteristic of translation in-variance through learning [33], the influence of noise in time series data is reduced. The pooling layers in CNNs filter out extraneous information or noise from the data, preserving only the essential features. This enhances the generalization ability of the anomaly detection model.

In this paper, we adopted GAF transformation to convert TS data into images, enabling more robust pattern recognition and improving anomaly detection. GAF is divided into the Gramian angular summation field (GASF) and the Gramian angular difference field (GADF) based on the transformation method. Both methods convert TS data into polar coordinates, mapping them onto a polar coordinate system based on angle and radius, and then transform them into images using a Gramian matrix. At this time, when there is a converted angle ϕ, it is divided into the GASF and GADF according to the summation/difference of the trigonometric function and is expressed as shown in Equations (1) and (2). According to these equations, the GADF highlights the rate of change in time series data and demonstrates superior performance compared with the GASF. This property is particularly advantageous in applications such as anomaly detection [34]. Therefore, in this paper, we utilize the GADF transformation method.

(1) $$\mathrm{GASF}=\cos\left(\phi_{i}+\phi_{j}\right)$$

(2) $$\mathrm{GADF}=\sin\left(\phi_{i}-\phi_{j}\right)$$

For the GADF transformation, the TS dataset *X* needs to be re-scaled to the range of [−1, 1], as shown in Equation (3). In this context, the Upper Bound (UB) is defined as the maximum value of the dataset $X=\left(x_{1},x_{2},\ldots ,x_{N}\right)$, the Lower Bound (LB) is defined as the minimum value and $\bar{X}$ represents the re-scaled dataset.

(3) $$\begin{array}{cc} {\bar{X}}_{i} & =\frac{x_{i}-\mathrm{UB}+\left(x_{i}-\mathrm{LB}\right)}{\mathrm{UB}-\mathrm{LB}}\in \left[-1,1\right] \end{array}$$

The re-scaled data, $\bar{X}$, obtained through Equation (3) can be represented in polar coordinates as shown in Equation (4). $t_{i}$ represents the timestamp of the scaled dataset $\bar{X}$, and *N* is the constant factor used to regularize the range of the polar coordinate systems.

(4) $$\left\{\begin{array}{c} \phi =\arccos\left({\bar{X}}_{i}\right);\;\phi \in \left[0,\pi \right]\\ r=\frac{t_{i}}{N};\;t_{i}\in N, \end{array}\right.$$

**Figure 4:**
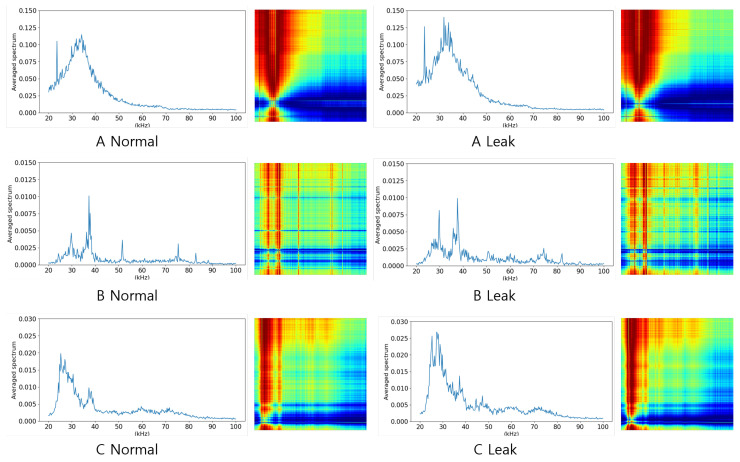
GADF translation results.

The polar coordinates can then be represented in matrix form, as shown in Equation (5), and *I* is the unit row vector $\left[1,1,\ldots ,1\right]$. Using this representation, images can be generated, and the transformed results of the pipeline leakage dataset in the proposed method are shown in Figure 4.

(5) $$\mathrm{GADF}=\sin\left(\phi_{i}-\phi_{j}\right)={\sqrt{I-{\bar{X}}^{2}}}^{T}\cdot \bar{X}-{\bar{X}}^{T}\cdot \sqrt{I-{\bar{X}}^{2}}$$

### 4.2. CycleGAN for Anomaly Generation

In this paper, we propose a method for generating abnormal data based on normal data from the target environment and detecting anomalies. Therefore, we adopted the CycleGAN model to generate abnormal data. CycleGAN achieves high performance in learning to transform between two image domains. CycleGAN is composed of two generators and two discriminators, where each generator is responsible for converting data from one domain to another. An important component is the loss of cycle consistency, a loss function that ensures that the converted data matches the original data when they are converted back to the original domain. More specifically, it is trained to ensure that, when normal data are transformed into abnormal data and translated back into normal data, the reconstructed normal data closely resembles the original normal data. This ensures that the generated data can be transformed into a new domain while maintaining the features of the original domain.

**Figure 5:**
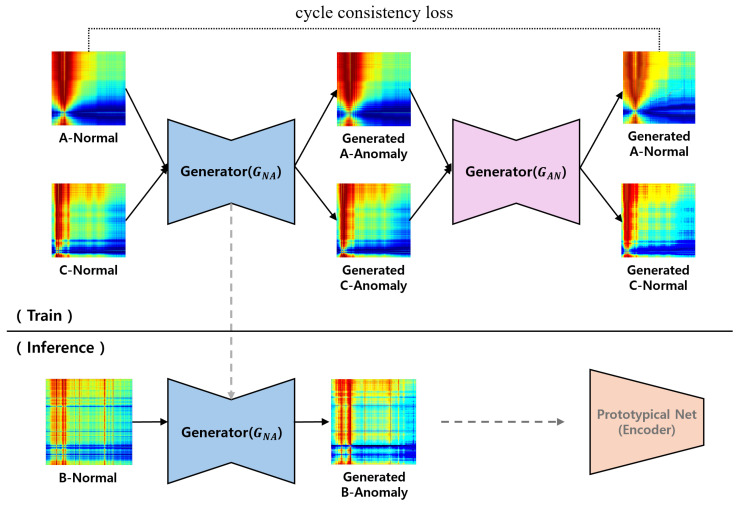
Training and inference process of anomaly generator using CycleGAN.

Therefore, in scenarios such as pipe leakage detection, where abnormal features are similar across various environments, this method proves effective in transforming normal data into abnormal data. The generator is trained on the features of pipe leakage using simulation data or normal and abnormal data from similar environments. Subsequently, the generator can be utilized for anomaly detection, even in situations where specific abnormal data from the target environment are lacking or unavailable. The overall training and generation process of the generators used in this paper is shown in Figure 5.

### 4.3. Prototypical Networks for Anomaly Detection

**Figure 6:**
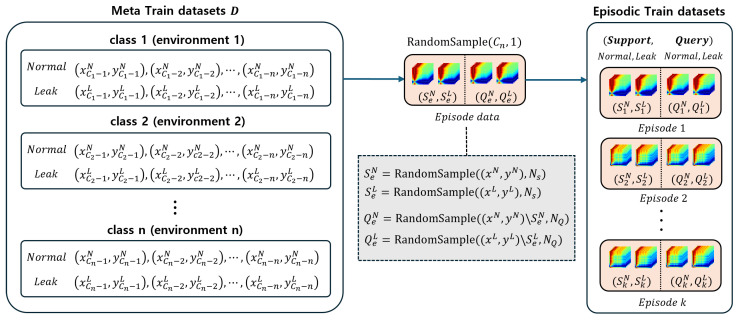
Dataset structure of episodic train.

In this paper, the PN was adopted to achieve anomaly detection using a small number of normal data from each environment and the leakage data generated in Section 4.2. The PN is a type of metric-based meta-learning model that classifies data by evaluating the similarity between the data to be inferred and the prototypes of each class within an embedding space. By repeatedly learning from environments and randomly sampled data, it is possible to achieve high classification performance, even with limited numbers of data. Furthermore, since classification is similarity-based, it can avoid potential problems that could result from differences between CycleGAN-generated anomaly data and the target domain’s anomaly data. To train the PN, it is necessary to create episodic training datasets using normal and leakage data from environments different from the inference environment. As shown in Figure 6, this process transforms training data with multiple episodes, and the classes of each episode are randomly sampled. Each episode is divided into support data and query data, both of which are randomly sampled and different from each other.

**Figure 7:**
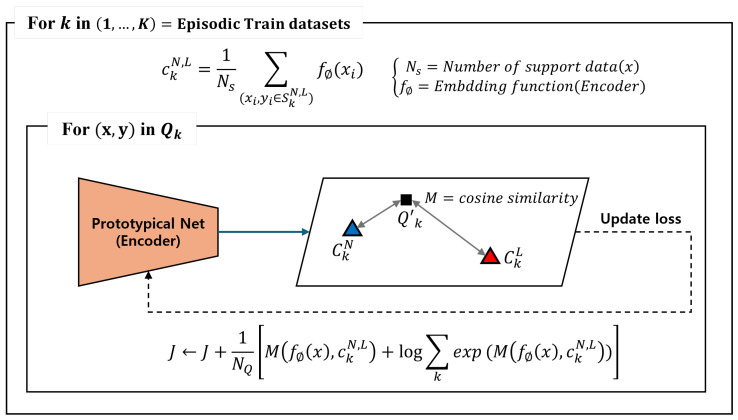
Training process of the prototypical network.

During training, the support data pass through the CNN encoder layers and are mapped into the embedding space. The mapped data are used to create prototypes by calculating the mean for each class in the matrix. Subsequently, the query data are also mapped into the embedding space using the same encoder, and the similarity between the prototypes is measured based on a specified metric. In this paper, a typical metric method of the PN includes Euclidean distance and cosine similarity, and cosine similarity is adopted. Based on the measured similarity, the model is updated, and iterating through multiple episodes ensures that the model can effectively classify normal and abnormal, even in new environments. During inference, a small number of data from the target domain are used to create the prototypes. After that, the inference data are classified into the class with the highest similarity to the prototypes for each respective class. The training process of the PN is described in Figure 7.

## 5. Experiment and Results

### 5.1. Results of GAF Transformation in Prototypical Networks

To validate the performance of the model using GAF image transformation, we compared it with a PN model that uses TS data as input. In this context, the comparison models, the PN (CNN, with GAF transformation) and the PN (MLP, *w*/*o* GAF transformation), applied the standard PN without any anomaly generation process. The environment of the training data for the experiment and the environment of the validation data were different. The validation results are shown as the average binary classification accuracy over 100 episodes, as presented in Table 3. Each episode consists of two-way (normal, leak) enabled data with 5 samples each to create a prototype and 15 query data samples for evaluating accuracy based on the similarity between the prototypes. The episode data for each class were sampled randomly, and cosine similarity was used to measure the similarity between the query data and the prototypes in the PN. Consequently, the PN (CNN, with GAF transformation) in all environments reported higher accuracy than the PN (MLP, *w*/*o* GAF transformation). The results demonstrate that the method of converting time series data into images maximizes the efficiency of PN-based anomaly detection. Based on these results, we conducted the experiments by applying GADF pre-processing to the training and test datasets of GAD-PN in Section 5.2. Additionally, to ensure fairness in the experiments, we applied the same pre-processing to all baseline models.

### 5.2. Results of GAD-PN

#### 5.2.1. Comparison with Zero-Shot Anomaly Classification

To verify the performance of the GAD-PN structure proposed in this paper, we compared it with a zero-shot-based CNN binary classification model. The structure of the CNN network was set to be the same as that of the encoder of the GAD-PN. The entire dataset from the training environment was used to train the CNN, and the binary classification accuracy of the entire dataset from the test environment was evaluated by the trained model.

In GAD-PN, the anomaly generator was trained on the entire dataset from the training environment, as described in Section 4.2. Through this process, the model learns the features of leaks using CycleGAN, enabling it to generate simulated leak data when provided with normal test data inputs. The PN for anomaly classification was trained on the meta-data in an episodic format, as explained in Section 4.3. For adaptation (learning) in the testing phase, only five randomly chosen normal support data points per episode were used for each environment. Using these support data, the pre-trained anomaly generator generated leak data, followed by the creation of normal and leak prototypes. After then, the binary classification accuracy was measured based on 100 episodes, each consisting of 15 normal (excluding the support data) and 15 leaks (not generated) query data samples. Table 4 shows that GAD-PN outperforms the zero-shot-based anomaly classification model in all environments. This demonstrates that our GAD-PN structure is robust, even in the absence of anomaly data. Moreover, it consistently maintained great performance over the CNN classifier (zero-shot) in all environments, indicating a higher generalization capability of the model.

#### 5.2.2. Comparison with Traditional Anomaly Detection

**Figure 8:**
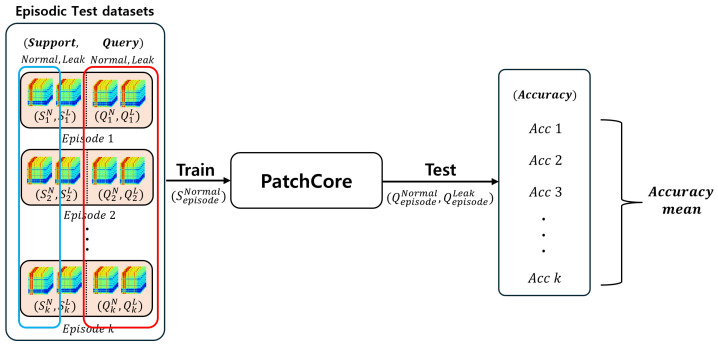
Inference process of PatchCore (few-shot).

In GAD-PN, while it receives a small number of normal data from the environment for inference, it is not a fully zero-shot anomaly classification. Therefore, we further compared it with a few-shot-based unsupervised anomaly detection that receives only normal data. For the comparison, we used PatchCore trained on few-shot data. PatchCore has shown high performance in general image anomaly detection, and, more recently, models based on it have shown higher performance. In this paper, we operated under the assumption of a limited number of training samples in the inference environment. Therefore, we conducted experiments using the same 100 episodes as GAD-PN in Section 5.2.1. In each episode, the model was trained with 5 normal support data samples and then tested with 15 normal and 15 leak query data samples, similar to the GAD-PN evaluation procedure. The accuracy of 100 episodes was averaged and the evaluation method is shown in Figure 8, and the results are shown in Table 5.

The experimental configuration of GAD-PN in Table 5 for GAD-PN is presented in Section 5.2.1, and the results are consistent. In comparison with PatchCore, a traditional unsupervised learning method, our method achieves higher accuracy: approximately 34.76% in environment A, 8.33% in environment B, and 48.03% in environment C. This demonstrates the effectiveness of GAD-PN in generating additional anomalous samples to train supervised learning-based classification when leakage characteristics in each environment are similar. Moreover, our method maintains consistent performance in all environments compared with conventional methods. This confirms that our approach enables more reliable anomaly detection in few-shot environments than traditional unsupervised anomaly detection methods.

#### 5.2.3. Comparison with Meta-Learning-Based Anomaly Detection

**Figure 9:**
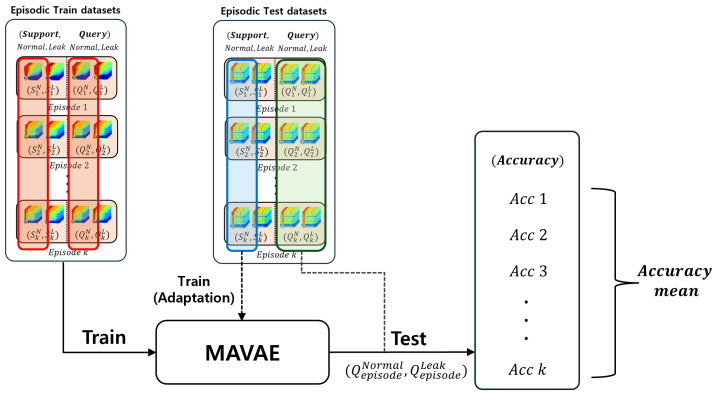
Inference process of MAVAE (few-shot).

MAVAE is a model that combines MAML and VAE for few-shot learning. It enables few-shot anomaly detection by learning initial parameters that rapidly adapt to new environments using metadata. In this paper, MAVAE learned metadata from different environments in an episode format. In the inference process, as outlined in Section 5.2.1, we adapted using 5 normal support data samples, the same as in GAD-PN, and evaluated based on 100 episodes consisting of 15 normal and 15 leak query data samples, as illustrated in Figure 9. The results shown in Table 6 demonstrate the average binary classification accuracy of the query data.

In comparison with MAVAE, an unsupervised learning method based on meta-learning, our approach achieves approximately 38.10% higher accuracy in environment A, 37.09% higher accuracy in environment B, and 34.45% higher accuracy in environment C. Even though MAVAE shows a consistent level of anomaly detection performance over the environment, it is difficult to form a normal distribution with only a small number of data, indicating that overall performance is inferior to our proposed model and PatchCore. This indicates that our method effectively overcomes the low-performance problems encountered when only a small number of normal data are available by forming an anomaly distribution through abnormal data generation.

## 6. Conclusions

In this paper, we aimed to realize anomaly monitoring and diagnosis in environments where real data collection is difficult, such as power plants and factories with inherent risk factors. Based on our proposed GAD-PN structure, we verify that anomaly monitoring and diagnosis are possible with only a small number of normal data. We also use a dataset consisting of ultrasonic measurements from pipe nozzles in three distinct environments for verification. The results show that GAD-PN achieves a classification accuracy of over 90% for both normal and leak conditions over all tested environments. Comparisons with zero-shot CNN classifiers, few-shot PatchCore, and MAVAE also demonstrate higher average accuracy. These results demonstrate that our work has the potential to enable more effective anomaly detection in limited anomaly detection environments where data collection is relatively challenging. In particular, we show that the GAD-PN structure can generate and learn anomaly data using only normal data, thus performing highly effective anomaly detection under various environmental conditions.

However, our proposed anomaly generation method with the GAD-PN structure assumes that anomaly features are similar between the training and deployment environments. Therefore, in environments with a range of anomaly categories, performance can be structurally unstable. For instance, an industrial environment may present a single sample with multiple types of anomalies, including broken or missing parts. In such cases, it becomes challenging to effectively learn anomaly features for all anomaly categories using a generative model. In future work, we will focus on generative or classification models to enhance robust anomaly detection for different anomaly cases.

## Figures and Tables

**Table 1:** International standard for pipe leak detection.

Standard	Class	Condition	
ASTM E 1002-11	Class II	Orifice diameter	≦0.2 mm
		Distance	5 m
		Pressure difference	0.7 atm
		Frequency	20 kHz∼100 kHz
		Leak rate	0.024 gpm

**Table 2:** Summary of data sampling in pipe leakage scenario.

State	A	B	C
Normal	981	363	995
Leak	981	778	1149

**Table 3:** Results of the GAF transformation-based standard PN.

Train	Test	Standard PN (MLP, *w*/*o* GAF Transformation)	Standard PN (CNN, with GAF Transformation)
A	C	59.70	98.57
B	C	68.63	99.97
A, B	C	74.17	100.00
B	A	59.00	94.63
C	A	55.73	94.77
B, C	A	56.77	97.33
C	B	63.93	98.90
A	B	71.63	99.40
C, A	B	60.67	99.50

**Table 4:** Comparison results with zero-shot anomaly classification.

Model	Train	Adaptation	Test	Accuracy
CNN Classifier	B, C	.	A	77.98
	C, A	.	B	49.85
	A, B	.	C	91.26
GAD-PN	B, C (episodic)	A (support)	A (query)	91.43
	C, A (episodic)	B (support)	B (query)	93.76
	A, B (episodic)	C (support)	C (query)	94.46

**Table 5:** Comparison results with PatchCore (few-shot).

Model	Train	Adaptation	Test	Accuracy
PatchCore	A (support)	.	A (query)	56.67
	B (support)	.	B (query)	85.43
	C (support)	.	C (query)	46.43
GAD-PN	B, C (episodic)	A (support)	A (query)	91.43
	C, A (episodic)	B (support)	B (query)	93.76
	A, B (episodic)	C (support)	C (query)	94.46

**Table 6:** Comparison results with MAVAE.

Model	Train	Adaptation	Test	Accuracy
MAVAE	B, C (episodic)	A (support)	A (query)	53.33
	C, A (episodic)	B (support)	B (query)	56.67
	A, B (episodic)	C (support)	C (query)	60.01
GAD-PN	B, C (episodic)	A (support)	A (query)	91.43
	C, A (episodic)	B (support)	B (query)	93.76
	A, B (episodic)	C (support)	C (query)	94.46

## Data Availability

Data are contained within the article.

## References

[1] Pang G., Shen C., Cao C., Hengel A.V.D. (2021). Deep learning for anomaly detection: A review. ACM Comput. Surv..

[2] Bhatt P.M., Malhan R.K., Rajendran P., Shah C.S., Thakar S., Yoon Y., Gupta S.K. (2021). Image-based surface defect detection using deep learning: A review. J. Comput. Inf. Sci. Eng..

[3] Nassif A.B., Talib M.A., Nasir Q., Dakalbab F.M. (2021). Machine learning for anomaly detection: A systematic review. IEEE Access.

[4] Wang Q.A., Dai Y., Ma Z.G., Ni Y.Q., Tang J.Q., Xu X.Q., Wu Z.Y. (2022). Towards probabilistic data-driven damage detection in SHM using sparse Bayesian learning scheme. Struct. Control Health Monit..

[5] Wang Q.A., Liu Q., Ma Z.G., Wang J.F., Ni Y.Q., Ren W.X., Wang H.B. (2024). Data interpretation and forecasting of SHM heteroscedastic measurements under typhoon conditions enabled by an enhanced Hierarchical sparse Bayesian Learning model with high robustness. Measurement.

[6] Li X., Cheng H.K., Qiu C.Z., Sichao T. (2022). Research on anomaly detection method of nuclear power plant operation state based on unsupervised deep generative model. Ann. Nucl. Energy.

[7] Yong S., Linzi Z. (2022). Robust deep auto-encoding network for real-time anomaly detection at nuclear power plants. Process Saf. Environ. Prot..

[8] Wang M., Lin T., Jhan K., Wu S. (2021). Abnormal event detection, identification and isolation in nuclear power plants using LSTM networks. Prog. Nucl. Energy.

[9] Zhang S., Ye F., Wang B., Habetler T. Few-Shot Bearing Anomaly detection via model-agnostic meta-learning. Proceedings of the International Conference on Electrical Machines and Systems (ICEMS).

[10] Feng T., Qi Q., Wang J., Liao J. Few-shot class-adaptive anomaly detection with model-agnostic meta-learning. Proceedings of the IFIP Networking Conference.

[11] Huisman M., Van Rijn J.N., Plaat A. (2021). A survey of deep meta-learning. Artif. Intell. Rev..

[12] Finn C., Abbeel P., Levine S. Model-agnostic meta-learning for fast adaptation of deep networks. Proceedings of the International Conference on Machine Learning (ICML).

[13] Snell J., Swersky K., Zemel R. Prototypical networks for few-shot learning. Proceedings of the Advances in Neural Information Processing Systems (NIPS).

[14] Zhu J.-Y., Park T., Isola P., Efros A.A. Unpaired Image-to-Image Translation Using Cycle-Consistent Adversarial Networks. Proceedings of the IEEE Conference on Computer Vision (ICCV).

[15] Roth K., Pemula L., Zepeda J., Schölkopf B., Brox T., Gehler P. (2022). Towards total recall in industrial anomaly detection. arXiv.

[16] Cohen N., Hoshen Y. (2020). Sub-image anomaly detection with deep pyramid correspondences. arXiv.

[17] Defard T., Setkov A., Loesch A., Audigier R. (2020). Padim: A patch distribution modeling framework for anomaly detection and localization. arXiv.

[18] Rabino P., Alliegro A., Borlino F.C., Tommasi T. (2023). OpenPatch: A 3D patchwork for Out-of-Distribution detection. arXiv.

[19] Mousakhan A., Brox T., Tayyub J. (2023). Anomaly detection with conditioned denoising diffusion models. arXiv.

[20] Bergmann P., Fauser M., Sattlegger D., Steger C. MVTec AD—A comprehensive real-world dataset for unsupervised anomaly detection. Proceedings of the IEEE Conf. on Computer Vision and Pattern Recognition (CVPR).

[21] Moon J., Noh Y., Jung S., Lee J., Hwang E. (2023). Anomaly detection using a model-agnostic meta-learning-based variational auto-encoder for facility management. J. Build. Eng..

[22] Kingma D.P., Welling M. Auto-encoding variational bayes. Proceedings of the International Conference on Learning Representations (ICLR).

[23] Sun H., Huang Y., Han L., Zhou C. (2023). Few-shot detection of anomalies in industrial cyber-physical system via prototypical network and contrastive learning. arXiv.

[24] Zhang H., Wu Z., Wang Z., Chen Z., Jiang Y.G. Prototypical residual networks for anomaly detection and localization. Proceedings of the IEEE Conference on Computer Vision and Pattern Recognition (CVPR).

[25] Wei Y., Jang-Jaccard J., Xu W., Sabrina F., Camtepe S., Boulic M. (2023). LSTM-Autoencoder-based anomaly detection for indoor air quality time-series data. IEEE Sens. J..

[26] Apostol E.-S., Truică C.-O., Pop F., Esposito C. (2021). Change point enhanced anomaly detection for IoT time series data. Water.

[27] Quan T.P., Lacey B., Peto T.E.A., Walker A.S. (2021). Health record hiccups—5526 real-world time series with change points labelled by crowdsourced visual inspection. GigaScience.

[28] Zhang G., Si Y., Wang D., Yang W., Sun Y. (2019). Automated detection of myocardial infarction using a gramian angular field and principal component analysis network. IEEE Access.

[29] Sun Y., Zhang J., Zhang Y. (2024). Swin transformer-based fluid classification using gram angle field-converted well logging data: A novel approach. Phys. Fluids.

[30] Song J., Lee Y.C., Lee J. (2023). Deep generative model with time series-image encoding for manufacturing fault detection in die casting process. J. Intell. Manuf..

[31] (2018). Standard Practice for Leaks Using Ultrasonics.

[32] Yeo D., Lee G., Lee J. (2020). Pipe leak detection system using wireless acoustic sensor module and deep auto-encoder. J. Korea Soc. Comput. Inf..

[33] Biscione V., Bowers J.S. (2021). Convolutional neural networks are not invariant to translation, but they can learn to be. J. Mach. Learn. Res..

[34] Feng M., Xu J. (2024). Enhancing non-destructive testing in concrete structures: A GADF-CNN approach for defect detection. J. Meas. Eng..

